# Fractional Subnanosecond Nd:YAG Laser Improves Matrix Architecture in Ultraviolet-Aged Facial Retaining Ligaments in Rats

**DOI:** 10.3390/ijms27188371

**Published:** 2026-09-20

**Authors:** Seyeon Oh, Geebum Kim, Myungjune Oh, Seung Min Oh, Min Seung Kim, Jae Ik Lee, Kuk Hui Son, Kyunghee Byun

**Affiliations:** 1 Functional Cellular Networks Laboratory, Lee Gil Ya Cancer and Diabetes Institute, Gachon University, Incheon 21999, Republic of Korea; 2 LIBON Inc., Incheon 22006, Republic of Korea; 3 Department of Anatomy & Cell Biology, College of Medicine, Gachon University, Incheon 21936, Republic of Korea; 4 Misogain Dermatology Clinic, Gimpo 10108, Republic of Korea; 5 Gangnam ON Clinic, Seoul 06129, Republic of Korea; 6 Inee Clinic, Seoul 06030, Republic of Korea; 7 Department of Thoracic and Cardiovascular Surgery, International Healthcare Center, Seoul National University Bundang Hospital, Seongnam 13620, Republic of Korea; 8 Department of Thoracic and Cardiovascular Surgery, Gachon University Gil Medical Center, College of Medicine, Gachon University, Incheon 21565, Republic of Korea; 9 Department of Health Sciences and Technology, Gachon Advanced Institute for Health & Sciences and Technology (GAIHST), Gachon University, Incheon 21999, Republic of Korea

**Keywords:** facial retaining ligament, photoaging, fractional subnanosecond laser, Nd:YAG, mechanotransduction

## Abstract

Facial retaining ligaments maintain soft-tissue position and support the lower face, but whether fractional subnanosecond Nd:YAG laser treatment can improve the matrix architecture of ultraviolet (UV)-aged ligament tissue remains unclear. We evaluated whether laser-associated matrix alteration was accompanied by coordinated changes in LATS1-YAP-IQGAP1-NFAT1 signaling and collagen type I-dominant remodeling. Male Sprague–Dawley rats were allocated to non-UV control, UV-aged, and UV-aged plus laser groups (*n* = 5 per group). After UV-aging induction, the laser group received a single fractional 1064 nm, 450 ps (subnanosecond) Nd:YAG treatment at 3.8 J/cm^2^ and 10 Hz using a 6 × 6 mm spot, with a measured pulse energy of 1.07 J and a calculated irradiance of 8.44 GW/cm^2^; 30 stacks, corresponding to 30 laser shots delivered to the same target area, were applied over 3 s. UV exposure increased cytosolic pLATS1/LATS1, cytosolic pYAP, and ELISA-detected IQGAP1-associated pYAP signals while reducing nuclear YAP; laser treatment partially reversed these changes. Concurrently, laser treatment decreased ELISA-detected IQGAP1-associated pNFAT1 signals, restored nuclear NFAT1 localization, and reduced cytosolic pNFAT1. Quantitative immunofluorescence, histomorphometric, and ultrastructural analyses showed restoration of the collagen type I signal and collagen type I/III ratio and improvements in collagen density, coherency, angular deviation, and collagen bundle diameter. Laser treatment restored the expression of tendon/ligament-lineage markers, including scleraxis, mohawk homeobox, and tenomodulin; UV-induced elevations in osteogenic markers (RUNX2, SP7, and ALPL) were not significantly attenuated. Overall, fractional subnanosecond Nd:YAG laser treatment partially improved the matrix architecture of UV-aged rat facial retaining ligaments. These exploratory findings support an association model linking laser-associated matrix remodeling with coordinated changes in LATS1-YAP-IQGAP1-NFAT1 signaling, lineage-marker recovery, and collagen type I-dominant realignment, but do not establish a causal signaling sequence or functional mechanical strengthening.

## 1. Introduction

Facial retaining ligaments are fibrous anchoring structures that connect the skin, superficial musculoaponeurotic system, facial fat compartments, deep fascia, and periosteum, thus maintaining the three-dimensional position of the cheek, periorbital, mandibular, and midfacial soft-tissue units [1,2,3,4]. In facial aesthetics, ligament integrity contributes to cheek projection, lid-cheek transition, nasolabial contour, jawline definition, and gravitational descent resistance [1,2,3,4]. Because these ligaments function as fixed anchoring points, weakening or lengthening of the ligamentous support system may permit soft-tissue descent; persistent tethering at fixed attachments might accentuate grooves and folds [2,3,4]. Facial rejuvenation cannot be understood solely as epidermal or dermal repair; the mechanical quality of deeper collagenous support structures must also be considered.

Ultraviolet (UV) irradiation is a major extrinsic driver of facial aging. In the skin, UV exposure induces matrix metalloproteinases through stress-responsive transcriptional pathways, promoting collagen and elastin degradation [5]. UV irradiation also suppresses type I procollagen synthesis by impairing transforming growth factor-β receptor II/Smad signaling, shifting the balance from extracellular matrix (ECM) synthesis toward degradation [6]. When collagen fibrils become fragmented, fibroblasts lose normal spreading and mechanical tension, further increasing matrix metalloproteinase-1 expression and perpetuating collagen fragmentation through c-Jun/activator protein-1 signaling [7]. Although most photoaging studies focus on the skin, ligament and tendon tissues are also dense collagenous matrices in which collagen quantity, collagen type I/III composition, fibril diameter, and fiber orientation determine tissue integrity and repair quality [8,9,10,11].

UV-aged ligaments are expected to contain a fragmented, poorly aligned, and mechanically abnormal collagenous microenvironment. Fractional 450 ps (subnanosecond) Nd:YAG lasers deliver ultrashort, high-peak-power pulses that can generate localized photomechanical microinjury, laser-induced optical breakdown, or cavitation-like microeffects, depending on wavelength, optical delivery method, pulse energy, and fluence [12,13]. Histologic and clinical studies of photoaged skin have shown that fractional ultrashort-pulse lasers induce dermal remodeling, although most evidence concerns the epidermis and dermis rather than facial retaining ligament tissue [13,14]. Consequently, we explored whether controlled fractional subnanosecond laser microstimulation was associated with remodeling of the aged ligament matrix and changes in mechanical cues perceived by resident ligament cells.

We selected Yes-associated protein (YAP) as the central mechanotransduction node because both YAP and transcriptional co-activator with PDZ-binding motif (TAZ) respond directly to physical cues, including ECM stiffness, cell spreading, and cytoskeletal tension [15]. In the canonical Hippo pathway, large tumor suppressor kinases 1 and 2 (LATS1/2) phosphorylate YAP to suppress its transcriptional activity [16]. Phosphorylation at serine 397 (Ser397)—equivalent to Ser381 in humans—is a key inhibitory signal. Although Ser397 phosphorylation ultimately triggers YAP degradation through the casein kinase 1 and β-transducin repeat-containing protein pathways, Ser127 phosphorylation is more directly responsible for YAP retention in the cytoplasm [16]. pYAP(Ser397) is best interpreted as a marker of LATS-mediated inhibition and degradation, rather than the primary driver of cytoplasmic retention.

Several prior observations support the possibility that light exposure, UV-induced matrix damage, and Hippo-YAP signaling are linked. Photobiomodulation with 670 nm light has been reported to inhibit Hippo signaling, induce YAP dephosphorylation and nuclear translocation, and require YAP for Müller glial regenerative responses, thereby providing proof of principle that light-based therapy can modulate YAP signaling in a biological repair context [17]. UVB exposure has been reported to alter Hippo pathway regulators in human epidermal keratinocytes; Hippo pathway activation has been implicated in UVB photosensitivity in lupus keratinocytes [18,19]. These observations were derived from non-ligamentous tissues and do not demonstrate that fractional subnanosecond Nd:YAG irradiation reduces LATS1 activity in facial ligament fibroblasts.

To extend this exploratory mechanistic framework, our hypothesis incorporates the cytoplasmic scaffold protein IQ motif-containing GTPase-activating protein 1 (IQGAP1), which directly binds YAP in previously studied systems and modulates its downstream transcriptional activity [20]. IQGAP1 is part of a cytoplasmic RNA-protein scaffold that includes the noncoding repressor of nuclear factor of activated T cells (NRON) and nuclear factor of activated T cells (NFAT) kinases; it regulates dephosphorylation and nuclear import of NFAT proteins [21]. Mechanosensing analysis of T cells showed that YAP interacts with IQGAP1, enhances IQGAP1-NFAT1 association, and requires YAP Ser397 for this coupling; a stiff microenvironment reduces IQGAP1-NFAT1 binding while increasing NFAT1 nuclear localization [22].

In tendon fibroblasts, scleraxis (SCX) and NFATc cooperate to regulate the pro-α1(I) collagen gene, linking NFAT activity to collagen type I transcription [23]. SCX also regulates tenomodulin (TNMD), a marker of mature tenocytes and ligamentocytes [24]. Mohawk homeobox (MKX) is another tendon/ligament-associated transcription factor that promotes tenogenic differentiation and has been implicated in anterior cruciate ligament biology [25,26]. Because ligament matrix is predominantly composed of collagen type I, whereas collagen type III is more closely associated with early repair or scar-like remodeling, the restoration of a collagen type I -dominant, aligned fibrillar matrix is more meaningful than nonspecific collagen accumulation [10,11].

On this basis, we hypothesized that fractional subnanosecond Nd:YAG laser irradiation would be associated with partial restoration of UV-aged facial retaining ligament matrix architecture and coordinated changes in pLATS1/LATS1, pYAP(Ser397), nuclear YAP, ELISA-detected IQGAP1-associated pYAP and pNFAT1 signals, nuclear NFAT1, tendon/ligament-lineage markers, and a collagen type I-dominant organization. This was evaluated as an association model rather than a proven sequential causal pathway. Given that YAP can also support osteogenic or heterotopic ossification programs in certain contexts, runt-related transcription factor 2 (RUNX2), Sp7 transcription factor/osterix (SP7), and alkaline phosphatase (ALPL) were evaluated as lineage-selectivity and safety-oriented markers, rather than as markers expected to decrease a priori [27,28]. This hypothesis represents an exploratory translation of evidence derived primarily from epidermal, retinal, and T cell models into facial retaining ligament biology. Accordingly, the cited studies provide biological plausibility and a parallel framework for hypothesis generation but do not establish a ligament-specific Hippo-YAP mechanism or an identical YAP-IQGAP1-NFAT1 pathway in facial ligament cells.

## 2. Results

### 2.1. Laser Treatment Reduced LATS1 Activation and Restored YAP Nuclear Availability in UV-Aged Facial Ligament Tissue

**Figure 1:**
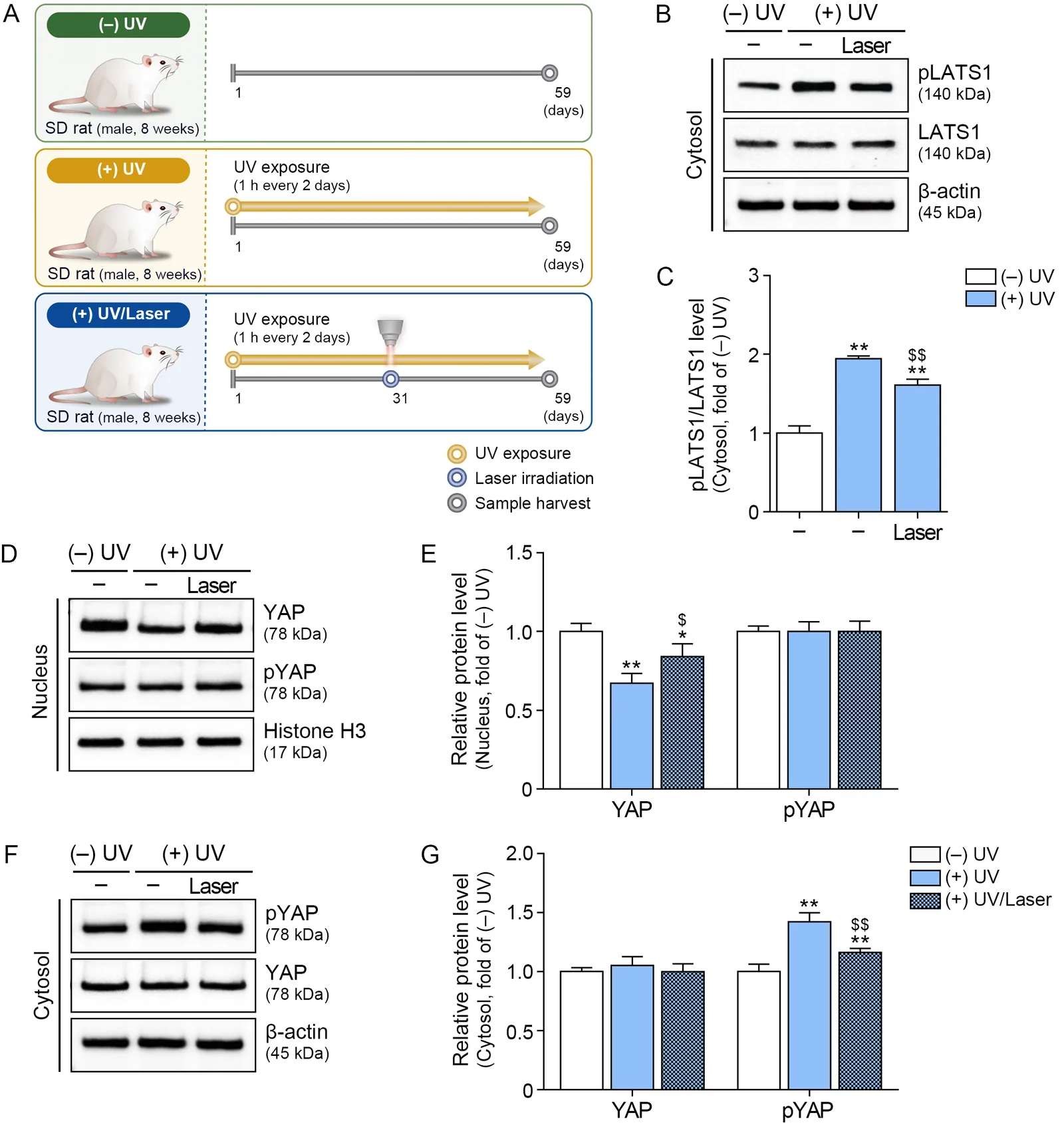
Laser irradiation modulates LATS1/YAP signaling in UV-exposed facial ligament tissue. (**A**) Schematic illustration of the experimental design. Rats were divided into three groups: (−)UV, (+)UV, and (+)UV/Laser. The (+)UV and (+)UV/Laser groups were exposed to UV irradiation for 1 h every other day for 30 days. On day 31, rats in the (+)UV/Laser group received a single laser treatment to the facial ligament region. Following laser application, UV exposure continued under the same conditions for an additional 28 days and facial ligament tissues were harvested on day 59. (**B**) Representative Western blot images of pLATS1 and LATS1 in cytosolic fractions. (**C**) Quantification of the cytosolic pLATS1/LATS1 ratio shown in (**B**). (**D**) Representative Western blot images of pYAP and YAP in nuclear fractions. (**E**) Quantification of nuclear YAP and pYAP levels shown in (**D**). (**F**) Representative Western blot images of pYAP and YAP in cytosolic fractions. (**G**) Quantification of cytosolic YAP and pYAP levels shown in (**F**). For Western blot analyses, Histone H3 and β-actin served as loading controls for nuclear and cytosolic fractions, respectively. Protein expression levels were normalized to Histone H3 or β-actin and expressed as fold changes relative to the (−)UV group. Data are presented as the mean ± standard deviation (*n* = 5 per group). Statistical significance was determined using the Kruskal–Wallis test followed by the Mann–Whitney U test for post hoc comparisons; *, *p* < 0.05 and **, *p* < 0.01, (−)UV vs. (+)UV or (+)UV/Laser; $, *p* < 0.05 and $$, *p* < 0.01, (+)UV vs. (+)UV/Laser. LATS1, large tumor suppressor kinase 1; pLATS1, phosphorylated LATS1; pYAP, phosphorylated yes-associated protein; UV, Ultraviolet; YAP, Yes-associated protein.

To assess the effect of laser irradiation on LATS1/YAP signaling in UV-exposed facial ligament tissue, rats were assigned to three experimental groups, as shown in Figure 1A. In cytosolic fractions, UV exposure increased the pLATS1/LATS1 ratio relative to that of the non-UV control group (Figure 1B,C). Laser treatment significantly reduced the pLATS1/LATS1 ratio compared with that of the UV-aged group, although it did not fully return to the non-UV control levels.

Nuclear YAP abundance decreased after UV exposure and was restored by laser treatment (Figure 1D,E), whereas nuclear pYAP did not significantly differ among groups in the overall quantification (Figure 1D,E). In cytosolic fractions, total YAP remained relatively unchanged across groups (Figure 1F,G); cytosolic pYAP increased after UV exposure and decreased after laser treatment (Figure 1F,G).

### 2.2. Laser Treatment Reduced IQGAP1-Associated pNFAT1 and pYAP Signals and Restored Nuclear NFAT1

**Figure 2:**
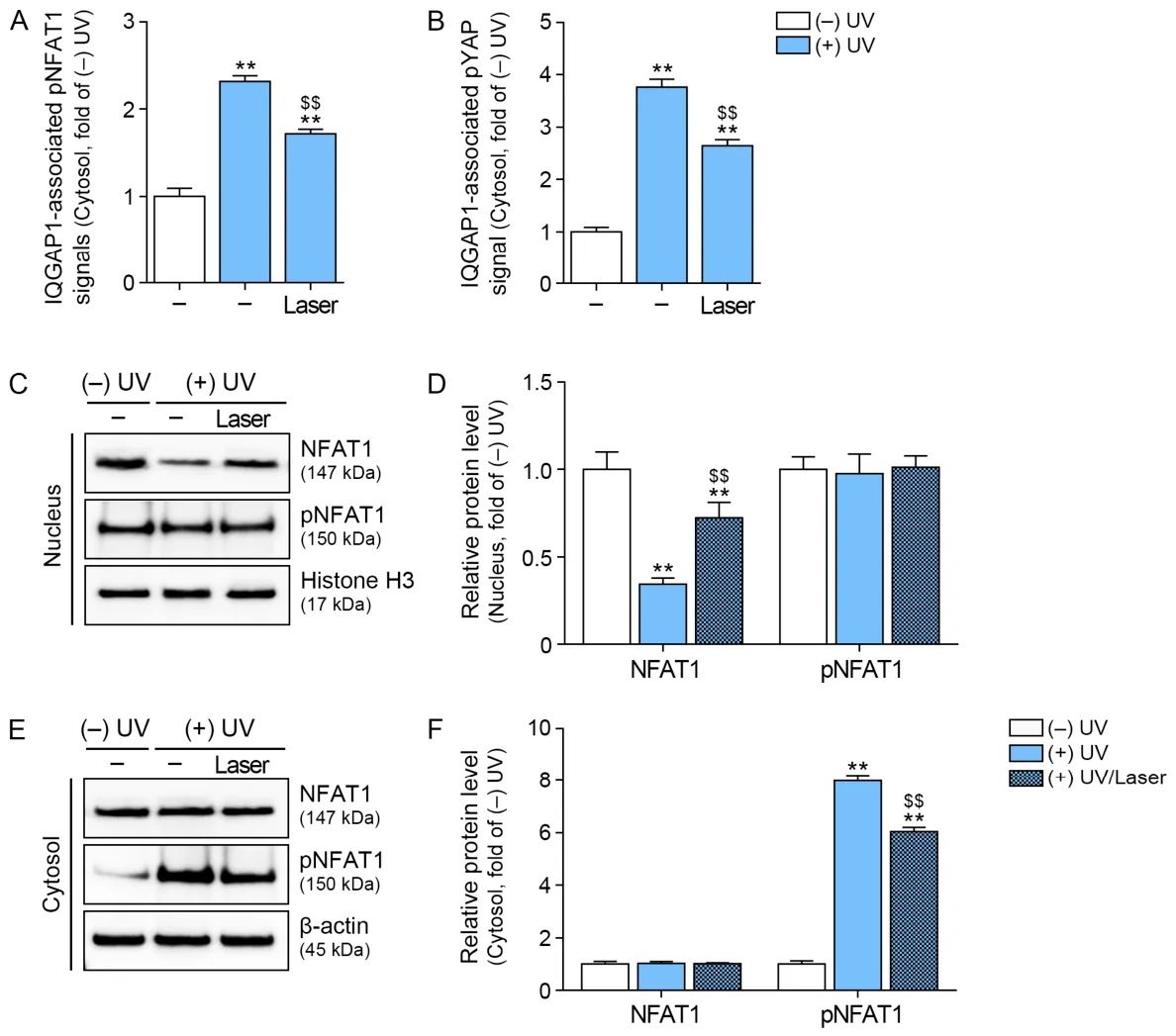
Laser treatment reduces ELISA-detected IQGAP1-associated pNFAT1 and pYAP signals, while restoring nuclear NFAT1 in UV-exposed facial ligament tissue. (**A**,**B**) Quantification of cytosolic IQGAP1-associated pNFAT1 and pYAP signals measured by sandwich ELISA, respectively. These assays indicate co-detection within IQGAP1-captured protein fractions and should not be interpreted as definitive evidence of direct binding or a ternary complex. (**C**) Representative Western blot images of pNFAT1 and NFAT1 in nuclear fractions. (**D**) Quantification of nuclear NFAT1 and pNFAT1 levels shown in (**C**). (**E**) Representative Western blot images of pNFAT1 and NFAT1 in cytosolic fractions. (**F**) Quantification of cytosolic NFAT1 and pNFAT1 levels shown in (**E**). For Western blot analyses, Histone H3 and β-actin served as loading controls for nuclear and cytosolic fractions, respectively. Protein expression levels were normalized to Histone H3 or β-actin and expressed as fold changes relative to the (−)UV group. Data are presented as the mean ± standard deviation (*n* = 5 per group). Statistical significance was determined using the Kruskal–Wallis test followed by the Mann–Whitney U test for post hoc comparisons; **, *p* < 0.01, (−)UV vs. (+)UV or (+)UV/Laser; $$, *p* < 0.01, (+)UV vs. (+)UV/Laser. IQGAP1, IQ motif-containing GTPase-activating protein 1; NFAT1, nuclear factor of activated T cells 1; pNFAT1, phosphorylated NFAT1; pYAP, phosphorylated yes-associated protein; UV, ultraviolet; YAP, Yes-associated protein.

Cytosolic IQGAP1-associated pNFAT1 signals were analyzed by sandwich ELISA to determine whether changes in the IQGAP1-associated pYAP signal were accompanied by corresponding changes in the IQGAP1-associated pNFAT1 signal. UV exposure significantly increased the ELISA-detected IQGAP1-associated pNFAT1 signal compared with that of the non-UV control group; laser treatment significantly decreased this signal relative to that of the UV-aged group (Figure 2A). The assay indicates co-detection within an IQGAP1-captured protein fraction but does not establish direct binding or a ternary protein complex.

UV exposure also increased the cytosolic ELISA-detected IQGAP1-associated pYAP (Ser397) signal. Laser treatment significantly decreased this association signal compared with that of the UV-aged group (Figure 2B).

In nuclear fractions, UV exposure substantially reduced total NFAT1, and laser treatment restored nuclear NFAT1 toward the non-UV control level. Nuclear pNFAT1 did not significantly differ among groups in the overall quantification (Figure 2C,D). In cytosolic fractions, UV exposure increased pNFAT1, whereas laser treatment reduced cytosolic pNFAT1 compared with that of the UV-aged group. Cytosolic total NFAT1 did not significantly differ among groups (Figure 2E,F).

### 2.3. Laser Treatment Restored Tendon/Ligament-Lineage Markers

**Figure 3:**
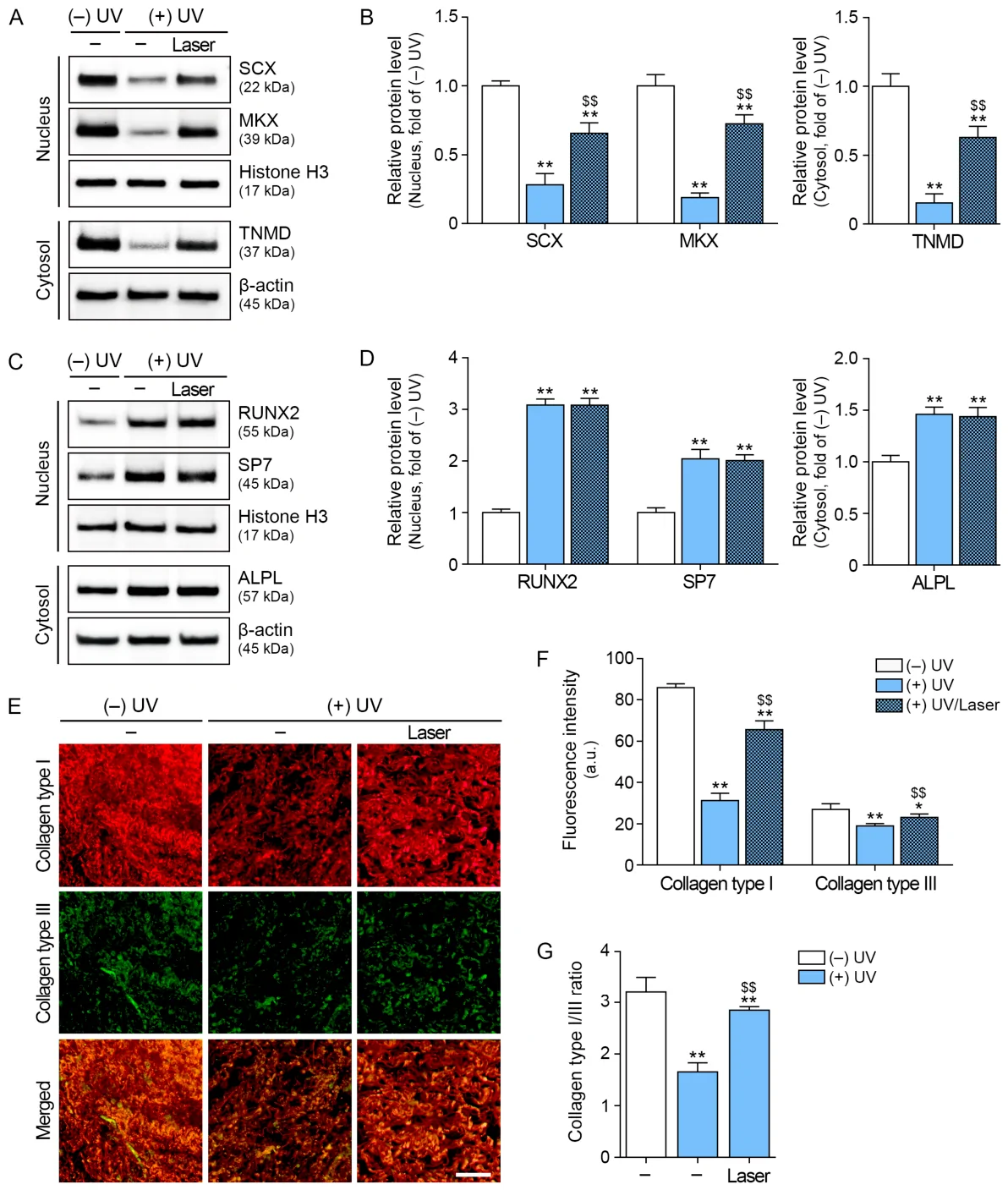
Laser treatment restores tendon/ligament-lineage markers and the collagen matrix phenotype in UV-exposed facial ligament tissue. (**A**) Representative Western blot images of nuclear SCX and MKX and cytosolic TNMD. (**B**) Quantification of nuclear SCX, nuclear MKX, and cytosolic TNMD protein levels shown in (**A**). (**C**) Representative Western blot images of nuclear RUNX2 and SP7 and cytosolic ALPL. (**D**) Quantification of nuclear RUNX2, nuclear SP7, and cytosolic ALPL protein levels shown in (**C**). For Western blot analyses, Histone H3 and β-actin served as loading controls for nuclear and cytosolic fractions, respectively. Protein expression levels were normalized to Histone H3 or β-actin and expressed as fold changes relative to the (−)UV group. (**E**) Representative immunofluorescence images of collagen type I and collagen type III in facial ligament tissue. Collagen type I is shown in red, collagen type III in green. Scale bar = 50 μm. (**F**) Quantification of collagen type I and collagen type III fluorescence intensities shown in (**E**). (**G**) Quantification of the collagen type I/III ratio, calculated for each sample using pair-matched collagen type I and collagen type III fluorescence intensities measured within the same region of interest. Data are presented as the mean ± standard deviation (*n* = 5 per group). Statistical significance was determined using the Kruskal–Wallis test followed by the Mann–Whitney U test for post hoc comparisons; *, *p* < 0.05 and **, *p* < 0.01, (−)UV vs. (+)UV or (+)UV/Laser; $$, *p* < 0.01, (+)UV vs. (+)UV/Laser. ALPL, alkaline phosphatase; MKX, mohawk homeobox; RUNX2, runt-related transcription factor 2; SCX, scleraxis; SP7, Sp7 transcription factor/osterix; TNMD, tenomodulin; UV, ultraviolet.

UV exposure considerably reduced nuclear SCX and MKX, as well as cytosolic TNMD, compared with levels in the non-UV control group (Figure 3A,B). Laser treatment significantly restored SCX, MKX, and TNMD levels compared with those of the UV-aged group.

UV exposure increased nuclear RUNX2 and SP7 and cytosolic ALPL compared with levels in the non-UV control group (Figure 3C,D). Laser treatment did not significantly reduce these markers relative to those of the UV-aged group.

Collagen type I/III co-immunofluorescence demonstrated that UV exposure reduced collagen type I signal intensity in facial ligament tissue (Figure 3E,F). Laser treatment increased the collagen type I signal toward the non-UV control level (Figure 3E,F). The collagen type III signal also decreased after UV exposure and increased after laser treatment (Figure 3E,F); the collagen type I/III ratio provided a more specific marker of ligament matrix phenotype. UV exposure reduced the collagen type I/III ratio, whereas laser treatment restored it toward the non-UV control level (Figure 3G).

### 2.4. Laser Treatment Improved Collagen Density, Fiber Alignment, Angular Dispersion, and Ultrastructural Bundle Diameter

**Figure 4:**
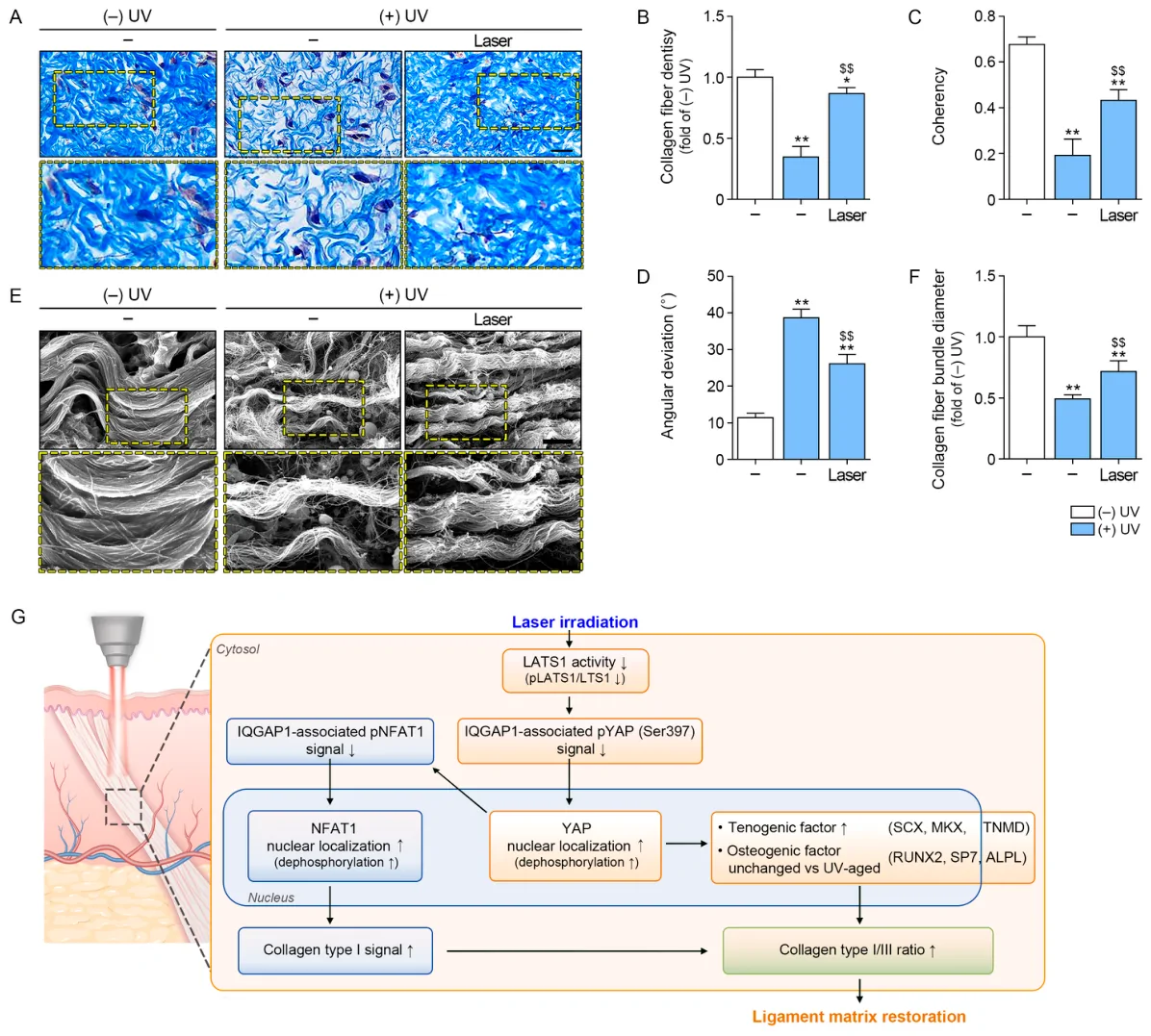
Laser treatment improves collagen density, fiber alignment, and ultrastructural organization in UV-aged facial ligament tissues. (**A**) Representative Masson trichrome-stained images of facial ligament tissue. Yellow dashed boxes indicate magnified regions shown below. Scale bar = 100 μm. (**B**) Quantification of collagen fiber density shown in (**A**). (**C**) Quantification of collagen coherency, reflecting directional fiber alignment shown in (**A**). (**D**) Quantification of angular deviation, reflecting dispersion of collagen fiber orientation angles shown in (**A**). (**E**) Representative SEM images of facial ligament collagen bundles. Yellow dashed boxes indicate magnified regions shown below. Scale bar = 5 μm. (**F**) Quantification of collagen bundle diameter shown in (**E**). (**G**) Proposed summary illustration of this study, illustrating a hypothetical association framework rather than a proven causal pathway. Connecting arrows indicate the proposed direction of signaling or downstream associations. Upward (↑) and downward (↓) arrows indicate increases and decreasesm repectively, in the indicaed activity, signal, expression, or nuclear localization, whereas “unchanged” indicates no significant change compared with the UV-aged group. ata are presented as the mean ± standard deviation (*n* = 5 per group). Statistical significance was determined using the Kruskal–Wallis test followed by the Mann–Whitney U test for post hoc comparisons; *, *p* < 0.05 and **, *p* < 0.01, (−)UV vs. (+)UV or (+)UV/Laser; $$, *p* < 0.01, (+)UV vs. (+)UV/Laser. ALPL, alkaline phosphatase; IQGAP1, IQ motif-containing GTPase-activating protein 1; LATS1, large tumor suppressor kinase 1; MKX, mohawk homeobox; NFAT1, nuclear factor of activated T cells 1; pLATS1, phosphorylated LATS1; pNFAT1, phosphorylated NFAT1; pYAP, phosphorylated yes-associated protein; RUNX2, runt-related transcription factor 2; SCX, scleraxis; SEM, scanning electron microscopy; SP7, Sp7 transcription factor/osterix; TNMD, tenomodulin; UV, ultraviolet; YAP, Yes-associated protein.

Masson trichrome staining showed dense, relatively organized collagen bundles in the non-UV control group (Figure 4A). UV-aged ligament tissue exhibited reduced collagen density, looser organization, and disrupted fiber architecture. Laser-treated UV-aged tissue displayed increased collagen density and improved collagen bundle organization compared with findings in the UV-aged group. Quantification confirmed that UV exposure greatly reduced collagen fiber density and that laser treatment significantly restored it (Figure 4B).

Quantitative orientation analysis provided additional evidence of architectural restoration. UV exposure decreased collagen coherency, indicating a loss of directional fiber alignment (Figure 4C). Laser treatment significantly increased coherency compared with that of the UV-aged group (Figure 4C). Conversely, UV exposure enhanced angular deviation, indicating greater dispersion of fiber orientation angles, whereas laser treatment reduced angular deviation (Figure 4D).

Scanning electron microscopy (SEM) further supported matrix restoration (Figure 4E). UV exposure reduced collagen bundle diameter and produced a more disorganized ultrastructural pattern. Laser treatment increased collagen bundle diameter compared with that of the UV-aged group (Figure 4F).

## 3. Discussion

The principal findings were that UV aging increased the pLATS1/LATS1 ratio, cytosolic pYAP, and ELISA-detected IQGAP1-associated pYAP and pNFAT1 signals while reducing nuclear YAP and NFAT1. UV aging also decreased the collagen type I signal and collagen type I/III ratio and impaired collagen architecture. Laser treatment partially reversed these changes: it reduced the pLATS1/LATS1 ratio, cytosolic pYAP, and IQGAP1-associated pYAP and pNFAT1 signals; restored nuclear YAP and NFAT1; restored the collagen type I/III ratio; and improved collagen density, coherency, angular deviation, and bundle diameter. Laser treatment also restored SCX, MKX, and TNMD expressions, whereas UV-induced elevations in RUNX2, SP7, and ALPL were not significantly attenuated. Taken together, these exploratory findings support an association model in which matrix architectural improvement is accompanied by coordinated changes in LATS1-YAP-IQGAP1-NFAT1-related readouts, tendon/ligament-lineage markers, and collagen type I-dominant realignment; they do not establish a sequential causal pathway.

The first mechanistic question is whether UV-aged ligament represents a mechanical microenvironment that can alter YAP signaling pathways. Our findings indicate that UV aging decreased collagen density, decreased coherency, enhanced angular deviation, reduced collagen bundle diameter, increased pLATS1/LATS1, increased cytosolic pYAP, increased ELISA-detected IQGAP1-associated pYAP signals, and decreased nuclear YAP. These observations are internally coherent because collagen fragmentation and loss of organized ECM architecture can reduce cellular spreading and mechanical tension, both of which are known to influence mechanotransduction [7,15]. Prior UV-focused studies support the plausibility of Hippo-YAP involvement: UVB can alter upstream Hippo regulators in human keratinocytes, and overactive Hippo signaling with LATS1/2 activation has been implicated in lupus photosensitivity [18,19]. However, those studies were performed in epidermal or disease-specific skin models, rather than rat facial ligament tissue; thus, they should be considered mechanistic support (not direct precedent) for the present model.

In our study, laser treatment reduced pLATS1/LATS1 and cytosolic pYAP while restoring nuclear YAP. These findings are consistent with a proposed model in which laser-associated matrix microstimulation alters mechanical cues sensed by ligament cells and is accompanied by reduced LATS1-associated YAP inhibition. YAP integrates ECM stiffness, cytoskeletal tension, and cell shape [15], and LATS-mediated YAP phosphorylation regulates YAP stability and activity in a site-specific manner [16]. A 670 nm photobiomodulation study demonstrated YAP dephosphorylation and nuclear translocation during mammalian retinal repair [17], but this non-ligament model is only a biological parallel. One hypothetical upstream route is that laser-induced photomechanical microinjury alters ECM organization and cell–matrix tension, thereby engaging cytoskeletal mechanotransduction and/or a mechanosensitive ion channel such as TRPV4 [15,29]. Calcium/calmodulin signaling can regulate LATS1-mediated inhibitory phosphorylation of YAP [30], providing a plausible link between mechanosensitive ion flux and Hippo signaling. Redox and inflammatory mediators remain additional untested upstream possibilities because oxidative stress and IL-6-family cytokine signaling can modulate Hippo-YAP activity in non-ligament systems [31,32]. However, the present experiments did not demonstrate direct LATS1 inhibition or measure any of these proposed upstream events.

The interpretation of pYAP(Ser397) deserves careful framing. In the present study, reduced cytosolic pYAP and reduced ELISA-detected IQGAP1-associated pYAP(Ser397) signals after laser treatment were direct experimental observations. These findings support the conclusion that the IQGAP1-associated Ser397-phosphorylated YAP pool decreased after laser treatment. However, pYAP(Ser397) should not be regarded as the sole determinant of YAP cytoplasmic retention. The C-terminal Ser397/Ser381 phosphodegron site is associated with LATS- and casein kinase 1-dependent YAP degradation, whereas Ser127 is more directly associated with 14-3-3-dependent cytoplasmic retention [16]. Accordingly, a more rigorous interpretation is that laser treatment attenuates LATS-associated inhibitory YAP phosphorylation, thus enhancing YAP nuclear availability, rather than establishing Ser397 dephosphorylation as the sole driver of nuclear translocation.

The inclusion of ELISA-detected IQGAP1-associated pNFAT1 signals extends the proposed association framework. UV-induced aging increased cytosolic IQGAP1-associated pNFAT1 signals and decreased nuclear NFAT1, whereas fractional laser treatment reduced these signals and restored nuclear NFAT1 levels. This pattern parallels established pathways in which IQGAP1 interacts with YAP and participates in a cytoplasmic scaffold regulating NFAT dephosphorylation and nuclear import [20,21]. Meng et al. reported YAP-dependent IQGAP1-NFAT1 coupling in a T cell mechanosensing model [22]. Because of the major cell-type and tissue-context differences, that model provides biological plausibility but does not establish an identical mechanism in ligament cells.

Accordingly, our results are best interpreted as supporting an IQGAP1-associated cytoplasmic restraint hypothesis. Laser treatment reduced ELISA-detected IQGAP1-associated pYAP and pNFAT1 signals while restoring nuclear YAP and NFAT1. This coordinated pattern is consistent with, but does not prove, a proposed sequence linking matrix reorganization, LATS-associated YAP phosphorylation, IQGAP1-associated protein pools, and NFAT1 nuclear recovery. The sandwich ELISA assays do not establish direct protein–protein binding or the existence of a single YAP-IQGAP1-NFAT1 ternary complex. Reciprocal co-immunoprecipitation, sequential immunoprecipitation, proximity ligation assays, YAP Ser397 mutagenesis, IQGAP1 knockdown, and NFAT/calcineurin inhibition are required to test these causal links.

The NFAT1 findings provide a plausible molecular context for collagen remodeling. NFAT proteins are classically regulated by phosphorylation-dependent cytoplasmic retention and calcineurin-dependent dephosphorylation, which permits nuclear import [21]. In tendon fibroblasts, SCX and NFATc cooperate to regulate the pro-α1(I) collagen gene [23]. In the present study, laser treatment restored nuclear NFAT1, SCX, and MKX expression levels; increased TNMD and collagen type I signals; and restored the collagen type I/III ratio. This coordinated expression pattern is consistent with the previously reported SCX/NFAT regulatory framework and a tendon/ligament-lineage repair signature. However, promoter occupancy and transcriptional activation were not directly assessed; ChIP or promoter–reporter experiments are required before direct SCX/NFAT regulation of collagen type I can be concluded in this model.

Although the coordinated recovery of tendon/ligament-lineage markers and collagen architecture supports a ligament-oriented remodeling response, the osteogenic marker profile warrants separate caution when considering the safety and translational implications of these findings. RUNX2, SP7, and ALPL were included because YAP biology is context-dependent and can support osteogenic or heterotopic ossification pathways in certain tissues [27,28]. UV exposure increased all three markers, and laser treatment did not significantly suppress these UV-associated elevations; importantly, it also did not further increase the measured markers beyond the UV-aged level. These expression data neither establish tissue mineralization nor exclude a longer-term calcification risk. Because calcium deposition, ectopic bone formation, and long-term mineralization were not directly evaluated, the present study cannot establish mineralization safety. Longer follow-up with Alizarin Red or Von Kossa staining, micro-computed tomography, and biochemical mineralization assays is required before aberrant calcification or heterotopic ossification can be excluded.

The histologic and ultrastructural findings indicate that molecular changes were accompanied by matrix-level restoration. Aged ligament tissue has been reported to exhibit fewer or atrophic fibrocytes, a looser collagen fibril arrangement, and reduced collagen fiber integrity [8]. Tendon aging similarly involves altered fibril morphology and changes to collagen and tendon-associated gene expression patterns [9]. In the present study, UV-aged facial ligaments showed reduced collagen density upon Masson trichrome staining and smaller collagen bundle diameter according to electron microscopy; laser treatment partially restored both. These structural changes are consistent with the recovery of SCX, MKX, TNMD, nuclear NFAT1, and a collagen type I-dominant matrix phenotype.

Coherency and angular deviation add an important quantitative dimension to matrix interpretation. Coherency is commonly derived from local orientation analysis using structure tensor-based image analysis and reflects the degree to which fibers share a dominant orientation; higher coherency indicates more directionally aligned collagen architecture [33,34]. Angular deviation, or fiber dispersion analysis, reflects the spread of collagen fiber orientation angles around the dominant direction; greater angular deviation indicates broader dispersion and diminished directional organization [33,35,36]. In the present study, UV aging decreased coherency and increased angular deviation, indicating that collagen fibers became less directionally organized and more dispersed. Laser treatment enhanced coherency and reduced angular deviation, indicating partial restoration of aligned collagen architecture. These findings are particularly relevant to ligament tissue because fiber alignment, rather than collagen abundance alone, is central to tensile matrix quality.

The collagen type I/III ratio further supports matrix-quality restoration. Native tendon and ligament matrices are predominantly composed of collagen type I; collagen type III is more prominent during early repair, remodeling, and scar-like matrix formation [10,11]. A nonspecific increase in collagen staining would not necessarily indicate ligament rejuvenation if the collagen remained disorganized or relatively collagen type III dominant. In our study, laser treatment restored the collagen type I signal and collagen type I/III ratio while also improving collagen density, coherency, angular deviation, and bundle diameter. These are quantitative image-based and ultrastructural measures, but they do not directly quantify total biochemical collagen content. Accordingly, the findings support architectural restoration rather than a definitive biochemical increase in total collagen. Hydroxyproline quantification, complementary biochemical collagen assays, quantitative polymerase chain reaction, proteomics, or second harmonic generation microscopy would strengthen future studies.

The novelty of the present study lies in its focus on facial retaining ligament tissue, rather than the epidermis or dermis alone. Current clinical management of ligament-related facial aging is primarily anatomical or procedural. Facelift and midcheek-lift procedures manipulate the superficial musculoaponeurotic system and facial spaces; they may release or reposition retaining ligaments to mobilize descended soft tissues [2,3]. These procedures can improve facial contour but do not directly reverse intrinsic ligament matrix aging. Platelet-rich plasma, dextrose prolotherapy, stem/progenitor cell therapy, and scaffold-based approaches have been investigated for tendons and ligaments outside the face; however, evidence remains heterogeneous and often indication-specific [37,38,39]. Fractional subnanosecond laser treatment conceptually differs because it aims to trigger endogenous remodeling of a collagenous support structure via transcutaneous energy delivery rather than through exogenous cells, biologics, or implanted matrices.

From a clinical perspective, structural improvement of the facial retaining ligament matrix could theoretically contribute to soft-tissue support; however, this possibility remains inferential. Histologic collagen restoration cannot be assumed to represent functional ligament strengthening without tensile testing, elastography, optical coherence tomography, ultrasound-based assessment of ligament thickness or stiffness, or objective facial contour analysis. Human retaining ligaments have specific layered relationships, depths, and biomechanical roles [1,2,3,4], whereas the rat model was developed as an experimental analog of age-related zygomatic-ligament change [40]. These distinct anatomical and loading contexts limit direct translation. Accordingly, this rat model should be regarded as a preclinical model of UV-associated collagenous ligament remodeling rather than a direct anatomical or biomechanical surrogate for human facial retaining ligaments. The findings are hypothesis-generating and do not establish immediate clinical efficacy, treatment depth, dosing, aesthetic benefit, or long-term safety in humans.

This exploratory proof-of-concept study has several important limitations. First, the sample size was small (*n* = 5 per group), limiting statistical precision and increasing the possibility of type II error; non-significant comparisons, particularly for the osteogenic markers, should not be interpreted as evidence of no biological effect. Second, the proposed laser-associated matrix-LATS1-YAP-IQGAP1-NFAT1 sequence was not causally tested. Third, the study did not directly measure tissue stiffness, strain, integrin/FAK/RhoA signaling, actomyosin tension, mechanosensitive ion-channel activity, calcium influx, calcineurin activity, reactive oxygen species, or inflammatory mediators. Fourth, IQGAP1-associated pYAP and pNFAT1 signals were assessed using sandwich ELISA rather than reciprocal co-immunoprecipitation or proximity assays. Fifth, the study did not include YAP inhibition or overexpression, LATS1/2 manipulation, IQGAP1 knockdown, NFAT/calcineurin inhibition, or SCX/NFAT promoter assays. Sixth, total collagen content was not directly quantified biochemically. Finally, RUNX2, SP7, and ALPL remained elevated after laser treatment, and long-term mineralization safety was not assessed.

Future prospectively powered studies should determine whether laser-associated matrix remodeling precedes or follows changes in LATS1-YAP signaling. Time-course experiments, mechanical testing, elastography, integrin-FAK-Src-RhoA analyses, actin cytoskeleton assessment, PIEZO1/TRPV4 and calcium/calcineurin assays, and redox modulation would help identify upstream events. Causal validation should include YAP/TEAD inhibition, LATS1/2 gain- and loss-of-function studies, IQGAP1 silencing, NFAT/calcineurin pathway modulation, reciprocal co-immunoprecipitation or proximity ligation, and ChIP or promoter–reporter assays. Biochemical collagen quantification and longer-term Alizarin Red or Von Kossa staining and micro-computed tomography should assess collagen content and mineralization safety. Translational studies should include human ligament imaging, appropriate dosimetry, mechanical endpoints, and long-term safety evaluation.

## 4. Materials and Methods

### 4.1. Subnanosecond Nd:YAG Laser System

Laser irradiation was performed using a commercially available picosecond Nd:YAG laser platform delivering 450 ps pulses, corresponding to a subnanosecond pulse duration (Picore Max; Bluecore Company Co., Ltd., Yangsan, Republic of Korea). The system operated at 1064 nm with a pulse duration of 450 ps and a repetition rate of up to 10 Hz. Laser energy was delivered through an articulated arm equipped with a dedicated handpiece and a Recurve SS tip (Bluecore Company Co., Ltd.). The laser beam was delivered with a spot size of 6 × 6 mm. The measured pulse energy was 1.07 J. Laser irradiation was performed at a fluence of 3.8 J/cm^2^, and the corresponding power density, calculated by dividing the fluence by the pulse duration of 450 ps, was 8.44 GW/cm^2^. The handpiece was used to direct irradiation to the designated facial ligament region. Detailed irradiation parameters available from the experimental record are described in Section 4.3.

### 4.2. Animals and Experimental Environment

Male Sprague–Dawley rats aged 7 weeks were purchased from Orient Bio (Seongnam, Republic of Korea). All experimental procedures were reviewed and approved by the Institutional Animal Care and Use Committee of the Lee Gil Ya Cancer and Diabetes Institute, Gachon University (Approval No. LCDI-2025-0014; approval date: 31 January 2025). Animal care and experimental procedures were conducted in accordance with the guidelines of the Association for Assessment and Accreditation of Laboratory Animal Care International and the ARRIVE guidelines.

Rats were maintained under controlled environmental conditions, including a temperature of 20–24 °C, relative humidity of 45–55%, and a 12 h light/dark cycle. Standard chow and water were provided ad libitum. Before the experiments, all animals underwent a 1-week acclimatization period.

### 4.3. Establishment of UV-Exposed Facial Ligament and Laser Treatment

After acclimatization, 15 rats were randomly allocated to three groups (*n* = 5 per group) [41]: (−)UV, (+)UV, and (+)UV/Laser. Animals in the (−)UV group did not receive UV exposure or laser treatment; animals in the (+)UV group received UV exposure but not laser treatment; animals in the (+)UV/Laser group received UV exposure followed by a single laser treatment. Before UV exposure, rats were anesthetized with inhaled isoflurane, and a 15 × 15 mm area of facial skin was shaved using an electric shaver (Haseong Electronics Co., Ltd., Seoul, Republic of Korea). UV-induced photoaging was established using a UV lamp (Sankyo Denki Co., Ltd., Tokyo, Japan; peak wavelength, 306 nm) positioned approximately 50 cm above the skin. The (+)UV and (+)UV/Laser groups were exposed to UV irradiation for 1 h every other day for a total of 30 days [42]. Based on the manufacturer-reported UV output and the irradiation geometry, the estimated UV dose was approximately 0.3 J/cm^2^ per 1 h of exposure [43].

On day 31, all animals were anesthetized, and the designated facial region was shaved as required for the procedure. Rats in the (+)UV/Laser group then underwent laser irradiation using a laser device equipped with a Recurve SS tip (Bluecore Company Co., Ltd.). The (−)UV and (+)UV groups underwent the same anesthesia and handling procedures but did not receive laser irradiation. Each laser-treated animal was positioned laterally, and the head was stabilized during irradiation. The Recurve SS tip was placed in direct contact with the skin surface overlying the anatomically identified facial ligament region. The tip was maintained perpendicular to the skin surface and kept in contact throughout irradiation. Laser treatment was performed at a wavelength of 1064 nm, a pulse duration of 450 ps, a fluence of 3.8 J/cm^2^, a repetition rate of 10 Hz, and a spot size of 6 × 6 mm. The measured pulse energy was 1.07 J, and the calculated power density was 8.44 GW/cm^2^. A total of 30 stacks, corresponding to 30 laser shots delivered to the same target area, were applied at 10 Hz, resulting in a total irradiation time of 3 s. The laser parameters were adapted from a manufacturer-provided clinical protocol currently used for human facial ligament treatment, in which 3.8 J/cm^2^ at 10 Hz and 30 stacked shots are applied to a single treatment site. For the rat model, these core irradiation parameters were maintained and applied to the smaller facial ligament target area.

After laser treatment, UV exposure was continued under the same conditions for an additional 28 days. The total experimental duration was 59 days. Two days after the final UV exposure, animals were euthanized, and facial ligament tissues were harvested from the designated anatomical region [40]. The collected tissues were used for subsequent histologic and molecular analyses. Outcome assessments were performed by investigators blinded to group allocation whenever feasible.

### 4.4. Extraction of Total, Nuclear, and Cytosolic Proteins

Total proteins were isolated from facial ligament tissues using EzRIPA buffer supplemented with protease and phosphatase inhibitors (ATTO Corporation, Tokyo, Japan). Samples were washed with cold phosphate-buffered saline (PBS), lysed on ice for 15 min, sonicated, and centrifuged at 14,000× *g* for 15 min at 4 °C. The resulting supernatants were collected as total protein extracts.

Nuclear and cytosolic proteins were prepared using a Nuclear and Cytoplasmic Extraction Kit (Thermo Fisher Scientific, Waltham, MA, USA), in accordance with the manufacturer’s protocol. Samples were washed with cold PBS and incubated in cytoplasmic extraction buffer on ice. After centrifugation at 12,000× *g* for 10 min at 4 °C, the supernatant was collected as the cytosolic fraction. The nuclear pellet was washed and incubated in nuclear extraction buffer on ice with intermittent vortexing. After centrifugation, the supernatant containing nuclear proteins was collected. Protein concentrations were determined using a bicinchoninic acid assay.

### 4.5. Preparation of Paraffin-Embedded Facial Ligament Tissue Sections

Facial ligament tissues were fixed in cold 4% paraformaldehyde (Sigma-Aldrich, St. Louis, MO, USA) for 72 h. Fixed tissues were washed with distilled water, placed in tissue cassettes, and processed through graded ethanol, xylene (Duksan, Ansan, Republic of Korea), and paraffin (Leica, Wetzlar, Germany) using an automated tissue processor (Leica). Paraffin-embedded blocks were prepared using an embedding system and sectioned at a thickness of 7 μm with a microtome (Leica). Tissue sections were mounted on coated slides and incubated overnight at 60 °C before staining.

### 4.6. Western Blot-Based Protein Expression Analysis

Protein expression was evaluated by Western blotting. Equal amounts of total, nuclear, or cytosolic protein were mixed with sample buffer (Thermo Fisher Scientific) and denatured at 70 °C for 10 min. Proteins were separated on 10% sodium dodecyl sulfate-polyacrylamide gels and transferred onto polyvinylidene difluoride membranes using a semidry transfer system.

After transfer, membranes were blocked with 5% nonfat dry milk in Tris-buffered saline containing 0.1% Tween-20 for 1 h at room temperature. Membranes were then incubated overnight at 4 °C with the primary antibodies listed in Table S1. After washing, membranes were incubated with horseradish peroxidase-conjugated secondary antibodies (Vector Laboratories, Burlingame, CA, USA) for 1 h at room temperature. Protein bands were detected using an enhanced chemiluminescence reagent and visualized with a ChemiDoc imaging system (Bio-Rad Laboratories, Hercules, CA, USA). Band intensities were quantified using ImageJ software version 1.53s (National Institutes of Health, Bethesda, MD, USA). β-actin was used as the loading control for total and cytosolic proteins; histone H3 was used as the loading control for nuclear proteins. Relative expression levels were normalized to the (−)UV group in each graph.

### 4.7. Sandwich ELISA-Based Assessment of IQGAP1-Associated Protein Signals

IQGAP1-associated protein signals were assessed by sandwich ELISA. Ninety-six-well plates were coated with capture antibodies diluted in carbonate-bicarbonate buffer (Sigma-Aldrich) and incubated overnight at 4 °C, then washed with PBS containing Tween-20. Next, they were blocked with 5% skim milk in PBS for 1 h at room temperature.

Protein samples were added to each well and incubated at 4 °C for 24 h. After the plates had been washed, detection antibodies against the corresponding binding partners were added and incubated overnight at 4 °C. Plates were then incubated with horseradish peroxidase conjugated secondary antibodies (Vector Laboratories) for 1 h at room temperature. The reaction was developed using 3,3′,5,5′-tetramethylbenzidine substrate and terminated with 2 N sulfuric acid. Absorbance was measured at 450 nm using a microplate reader (Thermo Fisher Scientific).

### 4.8. Immunofluorescence Analysis of Facial Ligament Tissue

Immunofluorescence staining was conducted to evaluate protein localization and collagen distribution in facial ligament tissue. Paraffin-embedded tissue sections were deparaffinized, rehydrated, and blocked with 3% normal goat serum in PBS for 1 h at room temperature. Sections were incubated overnight at 4 °C with primary antibodies diluted in blocking buffer, then washed with PBS. Next, they were incubated with fluorophore-conjugated secondary antibodies for 1 h at room temperature in the dark. Nuclei were counterstained with 4′,6-diamidino-2-phenylindole, and sections were mounted using an antifade mounting medium. Fluorescence images were acquired using a VS200 slide scanner (Olympus, Tokyo, Japan) and analyzed using OlyVIA software (version 4.2; Olympus).

### 4.9. Masson Trichrome Staining

Collagen deposition in facial ligament tissue was assessed by Masson trichrome staining. Tissue sections were incubated in Bouin solution (ScyTek, TRM-2; West Logan, UT, USA) at 60 °C for 1 h and rinsed with distilled water. Sections were sequentially stained with iron hematoxylin, Biebrich scarlet-acid fuchsin, phosphomolybdic-phosphotungstic acid, and aniline blue solutions. After staining, sections were washed, dehydrated, cleared, and mounted. Collagen-positive areas were quantified using ImageJ software version 1.53s.

### 4.10. SEM

For ultrastructural analysis, facial ligament tissue samples were fixed in Karnovsky fixative (2% glutaraldehyde and 2% paraformaldehyde in 0.1 M phosphate buffer, pH 7.4) for 24 h and washed with phosphate buffer. Samples were post-fixed with 1% osmium tetroxide, dehydrated through a graded ethanol series, and dried using a critical point dryer (EM CPD300; Leica). Dried samples were carbon-coated using an ion sputter coater (EM ACE600; Leica). Collagen fiber architecture was examined using a field-emission scanning electron microscope (MERLIN, Carl Zeiss Microscopy GmbH, Jena, Germany) operated at an accelerating voltage of 10 kV. Representative images were acquired at a magnification of 2.00 KX, with a typical capture time of approximately 11.3 s per image. Collagen bundle diameter was measured from scanning electron microscopy images using ImageJ software version 1.53s.

### 4.11. Statistical Analysis

Data are presented as the mean ± standard deviation. Group comparisons were performed using the Kruskal–Wallis test followed by the Mann–Whitney U test for post hoc comparisons. Statistical analyses were conducted using SPSS version 26 (IBM Corp., Armonk, NY, USA). A two-sided *p*-value < 0.05 was considered statistically significant. Exact two-sided *p*-values and corresponding effect sizes for pairwise Mann–Whitney U comparisons are provided in Table S2. Effect sizes were calculated as *r* = |Z|/$\sqrt{N}$, where *N* represents the total number of observations in the two groups being compared. Given the exploratory sample size, non-significant results were interpreted as statistically inconclusive rather than evidence of equivalence or absence of an effect.

Five animals per group were used in this exploratory animal study while minimizing animal use in accordance with ethical principles [41]. The study was not prospectively powered to establish equivalence or the absence of an effect. Consequently, the observed estimates should be regarded as exploratory, and larger prospectively powered studies are required for confirmatory inference.

## 5. Conclusions

Fractional subnanosecond Nd:YAG laser treatment partially improved matrix architecture in UV-aged facial retaining ligaments in rats. The findings support an association model in which matrix remodeling was accompanied by coordinated changes in pLATS1/LATS1, cytosolic pYAP(Ser397), nuclear YAP, ELISA-detected IQGAP1-associated pYAP and pNFAT1 signals, nuclear NFAT1, SCX, MKX, TNMD, and collagen type I-dominant architecture. Because causal perturbation, direct protein-binding validation, promoter occupancy, biochemical collagen quantification, mineralization assessment, and mechanical testing were not performed, the results do not establish a sequential signaling mechanism, functional strengthening, or immediate clinical applicability of the treatment.

## Data Availability

All data are contained within this article.

## Supplementary Materials

The following supporting information can be downloaded at https://www.mdpi.com/article/10.3390/ijms27188371/s1.
