# Supplementary material for: Fractional Subnanosecond Nd:YAG Laser Improves Matrix Architecture in Ultraviolet-Aged Facial Retaining Ligaments in Rats
Source: Int J Mol Sci. 2026 Sep 20;27(18):8371. doi: 10.3390/ijms27188371 (PMC13607763; doi:10.3390/ijms27188371)
Supplement: Supplementary file 1 [file ijms-27-08371-s001.zip › ijms-4499462-supplementary.pdf]

Table S1. List of antibodies used for ELISA, Western blot and immunofluorescence.

Antibody	Dilution ratio		
	**ELISA**	**Western blot**	**Immune fluorescence**
ALPL		1:200	
β -actin		1:1,000	
Collagen type I			1:100
Collagen type III			1:100
Histone H3		1:1,000	
IQGAP1	1:200		
LATS1		1:1,000	
MKX		1:2000	
NFAT1		1:500	
pLATS1		1:1,000	
pNFAT1	1:200	1:1,000	
pYAP	1:200	1:1,000	
RUNX2		1:2,000	
SCX		1:500	
SP7		1:1,000	
TNMD		1:750	
YAP		1:200	

Table S2. Exact p-values and effect sizes for pairwise statistical comparisons.

Figure	Outcome	Comparison	Exact p-value	Effect size (r)
Figure 1C	pLATS1/LATS1	(-)UV vs (+)UV	0.008	0.826
		(-)UV vs (+)UV/Laser	0.008	0.826
		(+)UV vs (+)UV/Laser	0.008	0.826
Figure 1E	Nuclear YAP	(-)UV vs (+)UV	0.008	0.826
		(-)UV vs (+)UV/Laser	0.016	0.760
		(+)UV vs (+)UV/Laser	0.016	0.760
	Nuclear pYAP	(-)UV vs (+)UV	1.000	0.033
		(-)UV vs (+)UV/Laser	1.000	0.033
		(+)UV vs (+)UV/Laser	1.000	0.033
Figure 1G	Cytosolic YAP	(-)UV vs (+)UV	0.310	0.363
		(-)UV vs (+)UV/Laser	1.000	0.033
		(+)UV vs (+)UV/Laser	0.421	0.297
	Cytosolic pYAP	(-)UV vs (+)UV	0.008	0.826
		(-)UV vs (+)UV/Laser	0.008	0.826
		(+)UV vs (+)UV/Laser	0.008	0.826

Figure 2A	IQGAP1-associated pNFAT1 signals	(-)UV vs (+)UV	0.008	0.826
		(-)UV vs (+)UV/Laser	0.008	0.826
		(+)UV vs (+)UV/Laser	0.008	0.826
Figure 2B	IQGAP1-associated pYAP signals	(-)UV vs (+)UV	0.008	0.826
		(-)UV vs (+)UV/Laser	0.008	0.826
		(+)UV vs (+)UV/Laser	0.008	0.826
Figure 2D	Nuclear NFAT1	(-)UV vs (+)UV	0.008	0.826
		(-)UV vs (+)UV/Laser	0.008	0.826
		(+)UV vs (+)UV/Laser	0.008	0.826
	Nuclear pNFAT1	(-)UV vs (+)UV	0.841	0.066
		(-)UV vs (+)UV/Laser	0.690	0.165
		(+)UV vs (+)UV/Laser	0.548	0.231
Figure 2F	Cytosolic NFAT1	(-)UV vs (+)UV	0.841	0.099
		(-)UV vs (+)UV/Laser	1.000	0.033
		(+)UV vs (+)UV/Laser	0.841	0.099
	Cytosolic pNFAT1	(-)UV vs (+)UV	0.008	0.826
		(-)UV vs (+)UV/Laser	0.008	0.826
		(+)UV vs (+)UV/Laser	0.008	0.826

Figure 3B	SCX	(-)UV vs (+)UV	0.008	0.826
		(-)UV vs (+)UV/Laser	0.008	0.826
		(+)UV vs (+)UV/Laser	0.008	0.826
	MKX	(-)UV vs (+)UV	0.008	0.826
		(-)UV vs (+)UV/Laser	0.008	0.826
		(+)UV vs (+)UV/Laser	0.008	0.826
	TNMD	(-)UV vs (+)UV	0.008	0.826
		(-)UV vs (+)UV/Laser	0.008	0.826
		(+)UV vs (+)UV/Laser	0.008	0.826
Figure 3D	RUNX2	(-)UV vs (+)UV	0.008	0.826
		(-)UV vs (+)UV/Laser	0.008	0.826
		(+)UV vs (+)UV/Laser	1.000	0.033
	SP7	(-)UV vs (+)UV	0.008	0.826
		(-)UV vs (+)UV/Laser	0.008	0.826
		(+)UV vs (+)UV/Laser	0.841	0.099
	ALPL	(-)UV vs (+)UV	0.008	0.826
		(-)UV vs (+)UV/Laser	0.008	0.826
		(+)UV vs (+)UV/Laser	0.841	0.099
Figure 3F	Collagen type I fluorescence intensity	(-)UV vs (+)UV	0.008	0.826
		(-)UV vs (+)UV/Laser	0.008	0.826
		(+)UV vs (+)UV/Laser	0.008	0.826
	Collagen type III fluorescence intensity	(-)UV vs (+)UV	0.008	0.826
		(-)UV vs (+)UV/Laser	0.032	0.693
		(+)UV vs (+)UV/Laser	0.008	0.826
Figure 3G	Collagen type I/III ratio	(-)UV vs (+)UV	0.008	0.826
		(-)UV vs (+)UV/Laser	0.008	0.826
		(+)UV vs (+)UV/Laser	0.008	0.826

Figure 4B	Collagen fiber density	(-)UV vs (+)UV	0.008	0.826
		(-)UV vs (+)UV/Laser	0.016	0.760
		(+)UV vs (+)UV/Laser	0.008	0.826
Figure 4C	Coherency	(-)UV vs (+)UV	0.008	0.826
		(-)UV vs (+)UV/Laser	0.008	0.826
		(+)UV vs (+)UV/Laser	0.008	0.826
Figure 4D	Angular deviation	(-)UV vs (+)UV	0.008	0.826
		(-)UV vs (+)UV/Laser	0.008	0.826
		(+)UV vs (+)UV/Laser	0.008	0.826
Figure 4F	Collagen fiber bundle diameter	(-)UV vs (+)UV	0.008	0.826
		(-)UV vs (+)UV/Laser	0.008	0.826
		(+)UV vs (+)UV/Laser	0.008	0.826
